# Evaluation of Four Endogenous Reference Genes and Their Real-Time PCR Assays for Common Wheat Quantification in GMOs Detection

**DOI:** 10.1371/journal.pone.0075850

**Published:** 2013-09-30

**Authors:** Huali Huang, Fang Cheng, Ruoan Wang, Dabing Zhang, Litao Yang

**Affiliations:** National Center for Molecular Characterization of Genetically Modified Organisms, SJTU-Bor Luh Food Safety Center, School of Life Science and Biotechnology, Shanghai Jiao Tong University, Shanghai, People’s Republic of China; Agriculture and Agri-Food Canada, Canada

## Abstract

Proper selection of endogenous reference genes and their real-time PCR assays is quite important in genetically modified organisms (GMOs) detection. To find a suitable endogenous reference gene and its real-time PCR assay for common wheat (*Triticum aestivum* L.) DNA content or copy number quantification, four previously reported wheat endogenous reference genes and their real-time PCR assays were comprehensively evaluated for the target gene sequence variation and their real-time PCR performance among 37 common wheat lines. Three SNPs were observed in the *PKABA1* and *ALMT1* genes, and these SNPs significantly decreased the efficiency of real-time PCR amplification. GeNorm analysis of the real-time PCR performance of each gene among common wheat lines showed that the *Waxy-D1* assay had the lowest M values with the best stability among all tested lines. All results indicated that the *Waxy-D1* gene and its real-time PCR assay were most suitable to be used as an endogenous reference gene for common wheat DNA content quantification. The validated *Waxy-D1* gene assay will be useful in establishing accurate and creditable qualitative and quantitative PCR analysis of GM wheat.

## Introduction

Although genetically modified organisms (GMOs) have been developed and marketed by many countries during the past two decades, controversies over safety issues have always been hot topics in public discussions. To protect consumers’ rights, many countries and regions have issued regulations and legislations to strengthen commercial GMO release and labeling management [1–3]. To effectively implement GMO labeling regulations, many efforts have been made to develop sensitive, accurate, and reliable methods for identification and quantification of GMOs, either gene-specific or event-specific [4–7].

For detection and quantification of GM contents, techniques based on nucleic acid analysis have been widely applied, including conventional PCR and TaqMan real-time PCR analysis [8]. The efficient and accurate quantification of host genome DNA copy numbers using endogenous reference genes is very important during the process [9]. In general, GM contents are expressed as mass/mass ratio or copy number ratio of GM over non-GM of the assayed organism. Therefore, endogenous reference genes and their real-time PCR assays are referred to as “golden standards” in GMO analysis [8]. One desirable endogenous reference gene and its real-time PCR assay should have three characteristics: species specificity, single or low copy number in the genome, and low heterogeneity among different lines [10]. In general, low heterogeneity is determined by minimum number of single nucleotide polymorphisms (SNPs) in the target DNA sequence and high PCR amplification performance among different lines [11,12].

To date, many endogenous reference genes and their real-time PCR assays have been developed for commercial GM crops, such as *Invertase I*, *zein*, *zSSIIb*, *adh* and *hmg-A genes* for maize; *HMG I/Y, FatA, CruA*, and *BnACCg8* genes for rapeseed; *PLD*, *SPS, GOS9*, and *ppi-PPF* genes for rice [8]. However, the increased number of reported endogenous reference genes has made it difficult to select the best candidate for a specific GMO analysis, and how to harmonize these endogenous reference genes is becoming not only important but also necessary in some cases. Recently, maize and rice endogenous reference genes and their real-time PCR assays were evaluated and harmonized employing 84 maize varieties and 58 rice varieties from different geographic and phylogenic origins, and the maize *zein* and/or z*SSIIb* gene, and the rice *SPS* gene assays were selected as best candidate in GM maize and rice analysis, respectively [11,12]. These reports demonstrated that proper evaluation and harmonization of different endogenous reference genes would be beneficial to host genome DNA quantification in routine laboratory analysis, proficiency testing, and GMO traceability.

Common wheat (*Triticum aestivum* L.) is one of the major staple food crops grown on more than 17% of the global cultivated land [13]. Wheat is the last major cereal crop to be genetically modified due to a complex genome structure, its recalcitrance to tissue culture, and challenges using *Agrobacterium* mediated gene transfer in this species [14,15]. Although no GM wheat has been commercialized so far, several GM wheat events, such as herbicide-tolerant and *Fusarium* -resistant GM wheat, are in the pipeline [16,17]. Many agricultural biotech companies (Monsanto, Bayer CropScience, and Syngenta, etc) also showed great interest in developing GM wheat events with traits such as drought tolerance, high yield, and more efficient phosphorus absorption [13]. For GM wheat detection and quantification, four different endogenous reference genes, *ACC1*, *PKABA1, ALMT1*, and *Waxy-D1*, as well as their corresponding real-time PCR assays have been previously reported [18–21]. The *ACC1* gene and its real-time PCR assay was suitable for the quantification of not only common wheat but also durum wheat, with the PCR performance verified in reactions employing 18 common wheat and 10 durum lines as templates [18]. The *PKABA1* real-time PCR assay was specific to wheat and barley, and one duplex real-time PCR assay with one set of primers and two TaqMan probes was developed for quantification of wheat and barley. However, it is difficult to develop one suitable *PKABA1* assay for GM wheat analysis with high specificity [19]. *ALMT1* real-time PCR assay was highly specific to common wheat and gave stable PCR performance in 15 lines [20]. The *Waxy-D1* real-time PCR assay was also highly specific to common wheat and generated similar PCR performance in 19 common wheat varieties [21].

To determine the most suitable endogenous reference gene and its real-time PCR assay for common wheat, we evaluated and harmonized the previously reported four endogenous reference genes and their assays according to the PCR target DNA sequence variations and real-time PCR performance among different hexaploid wheat lines. The results suggested that *Waxy-D1* gene and its real-time PCR assay was the most suitable because of its minimum sequence variation and best overall PCR performance among 37 tested hexaploid wheat lines. 

## Materials and Methods

### Plant Materials

Seeds of 43 wheat and relatives were kindly provided by the Institute of Crop Germplasm Resources, Chinese Academy of Agricultural Sciences (ICGR-CAAS), including 1 *Aegilops* *tauschii* , 1 *Aegilops* *spehoides* , 1 *Triticum* *urartu* , 1 *Triticum* *durum* (Cannizzo), 2 triticale ( *Xtriticosecale* *Wittmack*), and 37 hexaploid common wheat (*Triticum aestivum*) lines from different geographic and phylogenic origins. Brief description of the 43 wheat and relatives were listed in Table **S1** in File **S1**, and detailed information could be obtained from ICGR-CAAS website (icgr.caas.net.cn). We grouped these lines into four categories according to ploidy, which were diploid (genotype BB, DD and AA, including *Aegilops* *tauschii* , *Aegilops* *speltoides* *, and* *Triticum* *urartu* ), tetraploid ( *Triticum* *durum* , genotype BBAA), Hexaploid (genotype BBAADD, such as zhongmai 175), and octoploid triticale (genotype BBAADDRR, such as Xiaoyan 22 and Zhongguochun). In order to evaluate the PCR performance of the endogenous reference gene assays among different hexaploid common wheat lines, one common wheat was randomly selected (Jimai 19) for constructing of PCR standard curves and related analyses. All plants were grown in our greenhouse from germinated seeds, and fresh leaves were used for genomic DNA extraction.

### Genomic DNA Extraction and Purification

Genomic DNAs used for qualitative PCR and quantitative real-time PCR analysis were extracted and purified using Mini Plant Genomic DNA Extraction Kit (Shanghai Ruifeng Agro-tech Co. Ltd., Shanghai, China) according to manufacturer’s manual. The quantity and quality of the extracted DNA were evaluated using a Thermo Nanodrop 1000 spectrometer and 1.0% agarose gel electrophoresis. Each DNA preparation was firstly adjusted to a stock solution of 20 ng/µl, from which further dilutions were made.

### PCR Primers and Probes

According to the reported DNA sequence [18–21], PCR primers targeting the four endogenous reference genes were designed using Primer Premier 5.0 (PREMIER Biosoft Company, Canada) and used to amplify target gene fragment for sequencing. Two sets of primers and probes were used for real-time PCR analysis of the four endogenous reference genes. The first set was primers and probes described in previous reports [18–21]. The second set contained redesigned primers and probes based on the sequencing results of each amplified target DNA fragment to match the observed SNPs, including Q-Acc1-F2 and Q-ALMT1-F2 primers, Q-ALMT1-p2 and Q-PKABA1-p2 probes. All PCR primers and probes were synthesized by either Invitrogen Co., Ltd. or TaKaRa Biotechnology Co., Ltd and listed in Table **1**.

**Table 1:** Nucleotide sequences of the primers and probes used in this study.

**PCR type**	**Gene name**	**Primer/Probe name**	**Sequence（5’–3’)**	**Amplicon size**
Sequence PCR	*Acc1*	Seq-Acc1-F	TGCATCTGCGCTGTTTGT	184bp
		Seq-Acc1-R	AATGACTGAACACGACGACT	
	*ALMT1*	Seq-ALMT1-F	CCAAGGTGCTTAGGGATC	171bp
		Seq-ALMT1-R	CAAGAAGTGTTGCGGTGA	
	*Waxy-D1*	Seq-Waxy-F	CTATTGGTTCTCCGTGTTTG	263bp
		Seq-Waxy-R	GTCATCTGTCATTTCCTGGTT	
	*PKABA1*	Seq–*PKABA1*-F	AGGGAGATTTAGCGAGGAT	211bp
		Seq-*PKABA1*-R	TGTGCCGACAGTTGACTT	
Real-time PCR	*Acc1*	Q-Acc1-F	TGCCCATTGTCGGCCTTA	93bp
		Q-Acc1-R	GGGCAGATGGTTGGAATGC	
		Q-Acc1-p	FAM-TGCCTCGACAACACCATCGCTATCC-TAMRA	
		Q-Acc1-F2	TGCCCAT**C**GTCGGCCTTA	
	*ALMT1*	Q-ALMT1-F	AATGACTGTGCCGTCTCCAGT	95bp
		Q-ALMT1-R	ACAGAGCCGTGTTCTCTGCA	
		Q-ALMT1-p	VIC-CGTGAAAGCAGCGGAAAGCCTCAGA-TAMRA	
		Q-ALMT1-F2	A**C**TGACTGTGCCGTCTCCAGT	
		Q-ALMT1-p2	VIC-CGT***A***AAAGCAGCGGAAAGCCTCAGA-TAMR	
	*Waxy-D1*	Q-Waxy-F	GTCGCGGGAACAGAGGTGT	87bp
		Q-Waxy-R	GGTGTTCCTCCATTGCGAAA	
		Q-Waxy-p	FAM-CAAGGCGGCCGAAATAAGTTGCC-TAMRA	
	*PKABA1*	Q-PKABA1-F	CAAGTATGTCATAGAGATTTGAA	87bp
		Q-PKABA1-R	GTAACCGAAGTCACAAATCT	
		Q-PKABA1-P	FAM-TCGCACCTCGGCT-MGBNFQ	
		Q-PKABA1-p2	FAM-TCGCACCTCG***A***CT-MGBNFQ	

### Sequencing of amplified target DNA fragment

Target DNA fragments of *Acc1*, *ALMT1*, *Waxy-D1* and *PKABA1* in all 43 lines were amplified by PCR. The PCR reactions were carried on Verity thermal cycler (Applied Biosystems, Foster City, CA) with a 50 µl reaction volume. The PCR reaction contained 1× PCR reaction buffer for KOD-plus-DNA polymerase, 0.2 mM dNTPs, 1 mM MgCl_2_, 0.2 µM of each primer, 0.5 unit KOD-plus-DNA polymerase (TOYOBO Co. Ltd.), and 20 ng of extracted genomic DNA. The PCR program was: template denaturation at 94°C for 5 minutes; followed by 35 cycles of template denaturation at 94°C for 30 seconds, primer annealing at 58°C for 30 seconds, product extension at 68°C for 50 seconds; and a final PCR product extension at 68°C for 5 minutes. The PCR products were electrophoresed on 2% agarose gels to verify the correct size of each amplicon and target DNA fragment was excised from the gel and purified using DNA gel extraction kit (Axygen Bio-technology, Hangzhou, China). Each purified DNA was sequenced using ABI 3730 DNA Analyzer (BGI Co. Ltd., Shanghai, China). The sequences were blasted and aligned with target DNA sequence using Vector NTI Advance 10 software (Life Technologies, USA) to reveal the SNPs.

### Real-time PCR

Real-time PCR reactions were performed in optical 96-well PCR plates using ABI PRISM 7900HT sequence detection system (Applied Biosystems, Foster City, CA). The PCR reaction volume was 25 µl, containing 1 x universal amplification mix (Applied Biosystems, USA) and 5 µL template DNAs. 160 nM primers and 400 nM probe were used for *Acc1* and *ALMT1* assays, 80 nM primers and 200 nM probe were used for *Waxy-D1* gene assay, and 200 nM primers and 800 nM probe were used for *PKABA1* assay. All real-time PCR reactions were performed according to the program: 50°C for 2 minutes; 95°C for 10 minutes; and 45 cycles of 95°C for 15 seconds, 60°C for 60 seconds. Fluorescent signals were monitored at the extension step of 60°C in each cycle. For each sample test, each PCR reaction had 3 replicates and the experiment was repeated three times.

### Evaluation of Quantitative PCR Efficiency and *Ct* values

To evaluate the efficiency of quantitative PCR assay, standard curves were constructed using 5 serially diluted Jimai 19 genomic DNA solutions (10.0, 2.0, 0.4, 0.08, and 0.016 ng/µL). Optimal threshold lines were automatically determined using ABI 7900 software. Efficiency of each quantitative PCR was calculated using equation$\text{E}=10^{\left(-\frac{1}{m}\right)}-1$, where E was the PCR efficiency, *m* was the slope of the log transformed DNA quantities (x) versus *Ct* values (y) in equation y = m * log*x* + *b*. Real-time PCR amplification of each gene was performed at three template DNA concentrations (10.0, 1.0, and 0.2 ng/µL) with same PCR program. Measured *Ct* values under optimal threshold of their respective standard curves were recorded for all three DNA concentrations.

### Statistical Analysis

*Ct* values of PCR reactions using 50.0, 5.0 and 1.0 ng template DNA were calculated and analyzed for each endogenous reference gene. For each assay, *Ct* values from nine repeats were statistically analyzed for range, mean value, and standard deviation. The variations of each PCR assay among different lines were evaluated using GeNorm software (version 3.5, http://medgen.ugent.be/~jvdesomp/genorm/). GeNorm analysis is an algorithm widely used in evaluation and selection of suitable reference genes for gene expression studies. It has also been used in evaluating and harmonizing maize and rice endogenous reference genes and assays [11,12]. In this study, *Ct* values were transformed to relative quantities (Q) by *ΔCt* method (Q=*E* ^−*ΔCt*^), where E was the PCR efficiency of an assay using random selected DNA calibrator (Jimai 19), and *ΔCt* was the *Ct* difference between each individual cultivar and the calibrator (Jimai 19) at the same DNA concentration. The relative quantities were used as input data to automatically calculate the M value, which measured the corresponding average pairwise variation of a single reference gene against all other genes [22]. M value reflects the consistency of a target gene. Higher M value indicates lower consistency of a particular gene. For the three tested DNA concentrations, Q values of each reference gene were shown in a boxplot chart showing median, interquartile range (IQR), and the value ranges; and M values were displayed in a line connected dot plot according to the order of consistency of each gene. 

## Results

### Discovered SNPs in Real-time PCR Primer and Probe Annealing Regions of the Four Endogenous Reference Genes

The target DNA fragments of the four endogenous reference genes from all tested lines were amplified by conventional PCR and sequenced. The expected DNA fragment of *Acc1* gene could be amplified from all lines except *Aegilops* *tauschii* , *Aegilops* *speltoides* , and *Triticum* *urartu* ; expected DNA fragments of *ALMT1* and *Waxy-D1* genes were obtained from all lines except *Aegilops* *speltoides* *,* *Triticum* *urartu* , and *Durum wheat; and* expected *PKABA1* gene fragment was amplified from all lines except *Aegilops* *tauschii* and *Aegilops* *speltoides* (**Table S1 **in File **S1**). These results were the same as the previously reported results about the species specificity of these four genes [18–21], and failed amplifications were caused by genetic variation from all lines.

**Figure 1:**
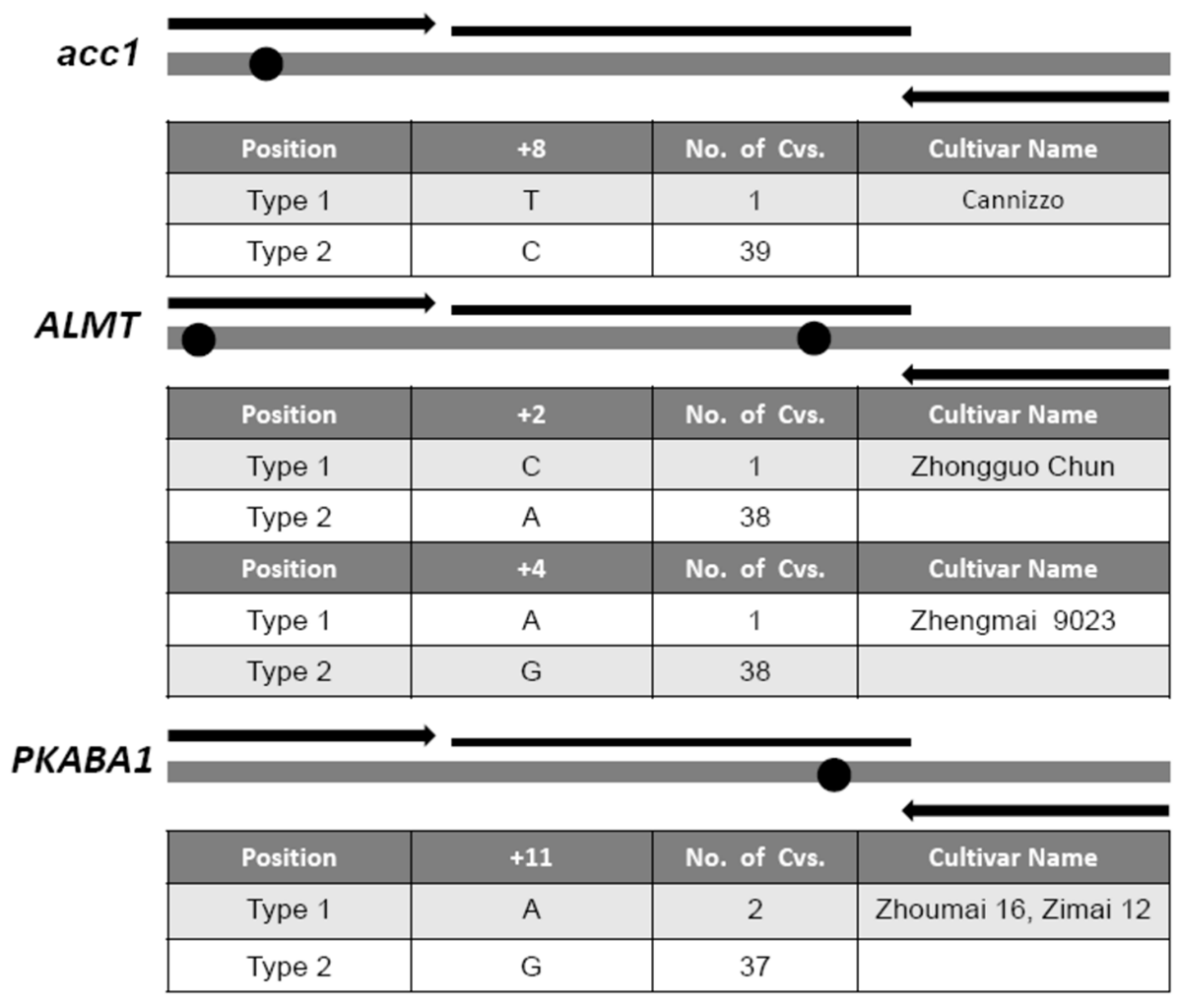
Detected SNPs within the amplified target DNAs of *Acc1*, *ALMT1*, and *PKABA1* gene. A SNP of C>T was found at the eighth nucleotide position of *Acc1* gene forward primer in durum wheat cultivar Cannizzo; A SNP of A>C was found at the second nucleotide position of *ALMT1* gene forward primer in common wheat cultivar Zhongguochun, and one SNP of G>A was at the fourth nucleotide position of *ALMT1* gene probe in common wheat cultivar Zhengmai 9023; A SNP of G>A was found at the eleventh nucleotide position of *PKABA1* gene probe in common wheat cultivars Zhoumai 16 and Zimai 12. No SNP was found in *Waxy-D1* target region.

After blastN and alignment analysis of the obtained DNA sequences from all line, SNPs were discovered in the real-time PCR target regions of *Acc1* gene, *ALMT1* gene, and *PKABA1* gene (Figure **1**). For *Acc1* gene, there was a SNP of C to T in durum wheat line Cannizzo, which was located at the eighth nucleotide from the 5’ end in the forward PCR primer. For *ALMT1* gene, a SNP of A to C was located at the second nucleotide from the 5’ end in the forward primer in common wheat Zhongguochun, and another SNP of G to A was located in the fourth nucleotide from the 5’ end of the probe in common wheat line Zhengmai 9023. For *PKABA1* gene, a SNP of G to A was found in the eleventh nucleotide from the 5’ end of the probe region in lines Zhoumai 16 and Zimai 12. No sequence variation was observed in the amplicon of *Waxy-D1* gene among all tested lines.

### Real-time PCR Efficiency and *Ct* Values

To establish efficient real-time PCR assays of the four wheat endogenous reference genes, five serially diluted genomic DNA solutions of common wheat line Jimai 19 (10.0, 2.0, 0.4, 0.08, and 0.016 ng/µL) were used to construct a PCR standard curve. PCR efficiencies of the four gene assays were between 0.920 and 1.045, indicating that all four real-time PCR assays had acceptable exponential efficiencies that were consistent with previously reported results except for the dramatical improvement of PCR efficiency in *PKABA1* assay [18–21]. The linear correlation (R^2^) values of the four constructed PCR standard curves were all above 0.990 (Table **2**), indicating these four assays were robust, with a wide dynamic range, and suitable for further quantitative analysis. Mean *Ct* values of each PCR assay were calculated from three repeats under identical threshold and listed in Table **S2** in File **S1**. These values could be used as references for further evaluation of the performance of each assay.

**Table 2:** PCR efficiencies and standard curve linearity of real-time PCR assays in their corresponding calibrator lines.

**Real-time PCR assay**	**Primers and probe**	**Calibrator**	**Linearity**	**PCR Efficiency**	**Standard curve**
*Waxy-D1*	Q-Waxy-F/R, Q-Waxy-p	Jimai 19	0.999	0.920	y = -3.5294*log χ+ 42.152
*ALMT1*	Q-ALMT1-F2/R, Q-ALMT1-p	Jimai 19	0.994	0.826	y = -3.8233*log χ + 45.699
		Zhongguochun	0.996	0.969	y = -3.3992*log χ + 43.813
	Q-ALMT1-F/R, Q-ALMT1-p2	Jimai 19	0.996	0.810	y = -3.8792*log χ + 47.025
		Zhengmai9023	0.994	0.950	y = -3.4484*log χ + 44.96
	Q-ALMT1-F/R, Q-ALMT1-p	Jimai 19	0.999	0.966	y = -3.4057*log χ + 42.216
		Zhongguochun	0.996	0.893	y = -3.6096*log χ + 44.114
		Zhengmai9023	0.997	0.868	y = -3.6842*log χ + 44.279
*PKABA1*	Q-PKABA1-F/R, Q-PKABA1-p	Jimai 19	0.998	1.045	y = -3.2178* log χ + 43.31
		Zhoumai16	0.997	1.075	y = -3.1546*log χ + 44.23
		Zimai12	0.982	0.885	y = -3.6328*log χ + 45.869
	Q-PKABA1-F/R, Q-PKABA1-p2	Jimai 19	0.995	0.804	y = -3.9012*log χ + 46.58
		Zhoumai16	0.997	0.933	y = -3.4933*log χ + 44.303
		Zimai12	0.997	0.964	y = -3.4108*log χ + 43.858
*Acc1*	Q-Acc1-F/R, Q-Acc1-p	Jimai 19	0.995	0.984	y = -3.361*log χ + 40.1
		Cannizzo wheat	0.997	0.808	y = -3.8891*log χ + 46.62
	Q-Acc1-F2/R, Q-Acc1-p	Jimai 19	0.999	0.864	y = -3.6971*log χ + 45.417
		Cannizzo wheat	0.998	0.928	y = -3.5079*log χ + 43.704

### Effects of the SNPs on Real-time PCR Assays

To evaluate the effects of the discovered SNPs in *Acc1*, *ALMT1* and *PKABA1* genes on their real-time PCR assays, PCR efficiency and *Ct* values in lines with detectable SNPs were analyzed. The recalculated PCR efficiencies of *Acc1*, *ALMT1*, and *PKABA1* genes in lines with SNPs were mostly below 0.90 and obviously lower than those in assays using lines containing no SNPs. For instance, the PCR efficiency of *ALMT1* gene in Zhongguochun and Zhengmai 9023 were 0.893 and 0.868, respectively; the PCR efficiency of *Acc1* gene in durum wheat Cannizzo was 0.808 (Table **2**).

*Ct* values of PCR assays of the four genes in different lines are listed in Table **3**. In *ALMT1* assay, the *Ct* values in Zhengmai 9023 (which had one SNP in probe region) using 50 ng, 5 ng, and 1 ng template genomic DNA were 29.34, 32.89 and 35.93, respectively. These *Ct* values were bigger than those in lines without SNPs, where the range of *Ct* values for *ALMT1* gene were 27.36-29.21, 30.77-32.65, and 32.72-35.74 for 50 ng, 5 ng, and 1 ng template genomic DNA, respectively. For *Acc1* assay, the *Ct* values in Cannizzo (with a SNP of C to T in the forward primer region) were 26.29, 29.48, and 31.78 at 50 ng, 5 ng, and 1 ng template DNA, and these *Ct* values were also bigger than the values in lines without SNPs, whose corresponding *Ct* range were 22.77-24.67, 25.97-28.49, and 27.83-30.5. Similar increase of *Ct* values was also observed for *PKABA1* assays in Zhoumai 16 and Zimai 12 which contained SNPs (Table **3**).

**Table 3:** *Ct* values of original assays and homogenous assays targeting on the SNPs in five wheat lines with SNPs.

**Real-time PCR assay**	**Primers and probe**	**Wheat cultivar**	***Ct* Values**		
			**50 ng**	**5 ng**	**1 ng**
***ALMT1***	Q-ALMT1-F/R and Q-ALMT1-p	Zhongguochun (A>C)	29.49	32.95	35.25
		Zhengmai 9023 (G>A)	29.34	32.89	35.93
		Cultivars without SNPs	27.36-29.21	30.77-32.65	32.72-35.74
	Q-ALMT1-F2/R and Q-ALMT1-p	Zhongguochun (A>C)	27.90	31.16	33.22
	Q-ALMT1-F/R and Q-ALMT1-p2	Zhengmai 9023 (G>A)	28.14	31.40	32.52
***PKABA1***	Q-PKABA1-F/R and Q-PKABA1-p	Zhoumai 16 (A>G)	30.29	33.39	35.40
		Zimai 12 (A>G)	30.27	33.54	35.41
		Cultivars without SNPs	27.66-29.93	30.66-32.66	32.49-34.86
	Q-PKABA1-F/R and Q-PKABA1-p2	Zhoumai 16 (A>G)	28.60	31.70	34.17
		Zimai 12 (A>G)	28.91	32.17	34.64
***Acc1***	Q-Acc1-F/R and Q-Acc1-p	Cannizzo wheat (C>T)	26.29	29.48	31.78
		Cultivars without SNPs	22.77-24.67	25.97-28.49	27.83-30.5
	Q-Acc1-F2/R and Q-Acc1-p	Cannizzo wheat (C>T)	24.39	27.78	29.19

### Confirmation of the SNP Effects on Real-time PCR Efficiencies

In order to confirm that the increased *Ct* values and lowered PCR efficiencies were indeed caused by the SNPs, PCR primers (Q-*ALMT1*-F2 for *ALMT1* assay and Q-*Acc1*-F2 for *Acc1* assay) and probes (Q-*ALMT1*-F2 for *ALMT1* assay, Q-*PKABA1*-p2 for *PKABA1* assay) that had matched nucleotides at the SNP sites were designed and used to test for *Ct* values and PCR efficiencies. PCR efficiencies calculated from reconstructed standard curves of *Acc1*, *ALMT1*, and *PKABA1* genes using new primers or probes are listed in Table **2**. The new PCR efficiencies were 0.928 for *Acc1* in Cannizzo, 0.969 for *ALMT1* in Zhongguochun, 0.950 for *ALMT1* in Zhengmai 9023, 0.933 for *PKABA1* in Zhoumai16, and 0.964 for *PKABA1* in Zimai12. All PCR efficiencies were obviously increased from those in real-time PCR assays using primers and probes containing unmatched SNPs (where PCR efficiencies were mostly below 0.90). On the contrary, when the new primer/probe sets adjusted for SNPs in some lines were used for Jimai 19, where the primer/probe had mismatched SNPs, the PCR efficiencies decreased dramatically (Table **2**), indicating that the mismatched SNPs caused the decrease of real-time PCR efficiency.

*Ct* values of the new homogenous PCR assays were also calculated using the same threshold (Table **3**). For *ALMT1* assay in Zhongguochun using Q-*ALMT1*-F2/R and Q-*ALMT1*-p, the *Ct* values were 27.90, 31.66, and 33.22 for 50 ng, 5 ng, and 1 ng template DNA. For *ALMT1* assay using Q-*ALMT1*-F/R and Q-*ALMT1*-p2, the *Ct* values were 28.14, 31.40, and 32.52 for 50 ng, 5 ng, and 1 ng genomic DNA in Zhengmai 9023. Similarly lowered *Ct* values were also observed for the other two genes containing SNPs (Table 2). The results demonstrated that real-time PCR *Ct* values could be lowered into normal ranges when SNPs were eliminated from the primer/probe region, indicating the direct effect of SNPs on PCR amplified efficiency.

### Comparison of the Real-time PCR Performance among the Four Endogenous Reference Genes

**Figure 2:**
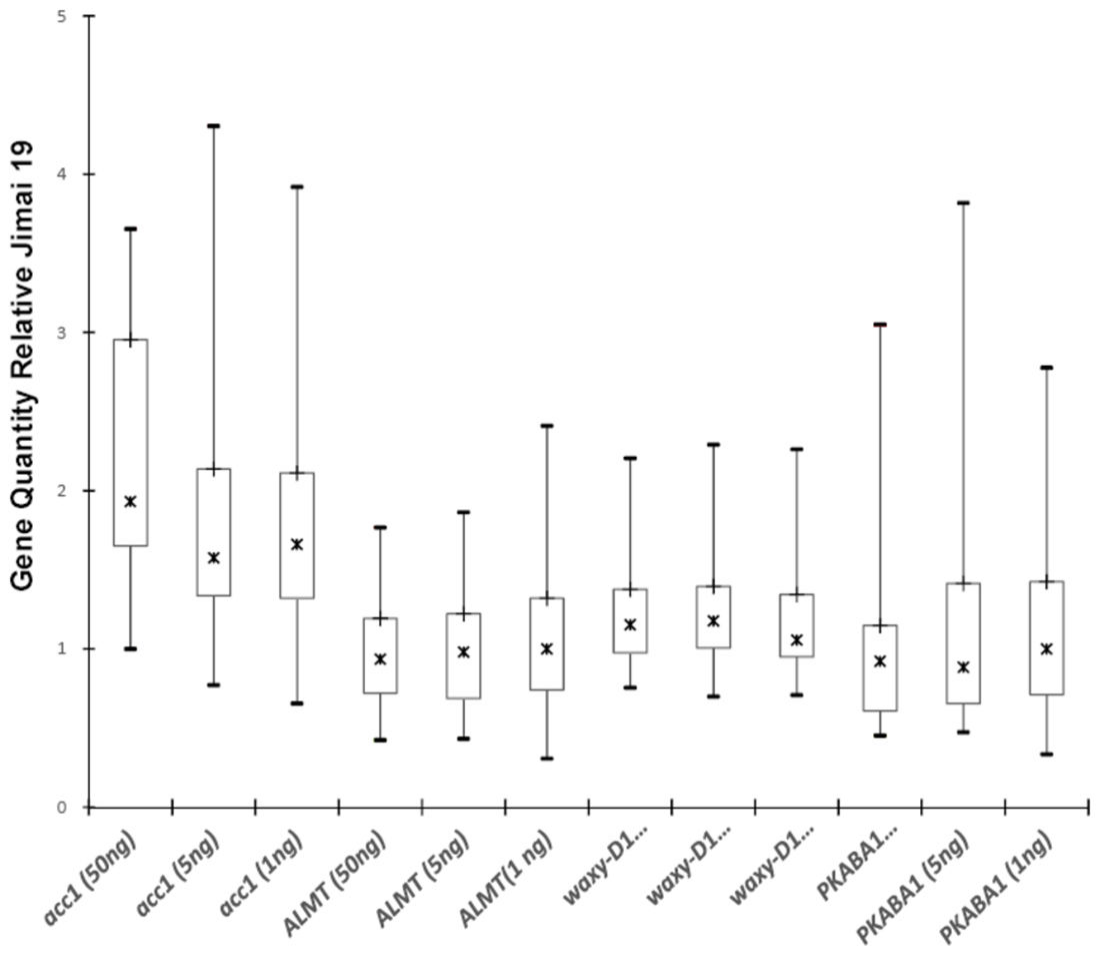
Quantity of the reference genes in three sets of assays as compared with the Jimai 19 calibrator (Q = E^ΔCt^). Boxplot shows median, interquartile and range.

To evaluate the performance of the four real-time PCR assays, 37 hexaploid wheat lines and 2 octoploid triticale lines were tested in this study. *Ct* values obtained from all 39 lines with the same threshold were used for statistical analysis and comparison (**Table S2 **in File **S1**). The variability of each real-time PCR assay was first evaluated using *Ct* values across all 39 tested lines. The assay with the lowest variation (judged by standard deviation, SD) of *Ct* values was the assay for *Waxy-D1* gene (0.40, 0.42, and 0.43 using three different amount of template genomic DNAs). The *PKABA1* assay had the largest *Ct* value variation in all 39 lines (0.70, 0.70, and 0.68 using three different amount of template genomic DNAs) (Table **4**). To evaluate the consistency of each of the four endogenous reference gene real-time PCR assays, *Ct* values were converted to relative quantitative values. Boxplot charts were constructed to show median and interquartile range of the relative quantitative values (Figure **2**). Based on the chart, the *ALMT1* and *Waxy-D1* gene assays appeared relatively consistent among all tested lines.

**Table 4:** *Ct* values of the four common wheat reference genes in all wheat lines.

**Gene name**			**50 ng**				**5 ng**				**1 ng**				
	***Ct*_range_**	***Ct*_min_**	***Ct*_max_**	**Mean *Ct*±SD**	***Ct*_range_**	***Ct*_min_**	***Ct*_max_**	**Mean *Ct*±SD**	***Ct*_range_**	***Ct*_min_**	***Ct*_max_**	**Mean *Ct*±SD**
***ALMT1***	2.13	27.36	29.49	28.49±0.52	2.18	30.77	32.95	31.84±0.54	3.21	32.72	35.93	34.42±0.67
***PKABA1***	2.63	27.66	30.29	28.68±0.70	2.88	30.66	33.54	31.65±0.70	2.92	32.49	35.41	34.00±0.68
***Acc1***	1.90	22.77	24.67	23.84±0.47	2.52	25.97	28.49	27.14±0.55	2.67	27.83	30.5	29.26±0.59
***Waxy-D1***	1.68	29.01	30.69	29.68±0.40	1.86	32.27	34.13	33.70±0.42	1.82	34.64	36.46	35.36±0.43

**Figure 3:**
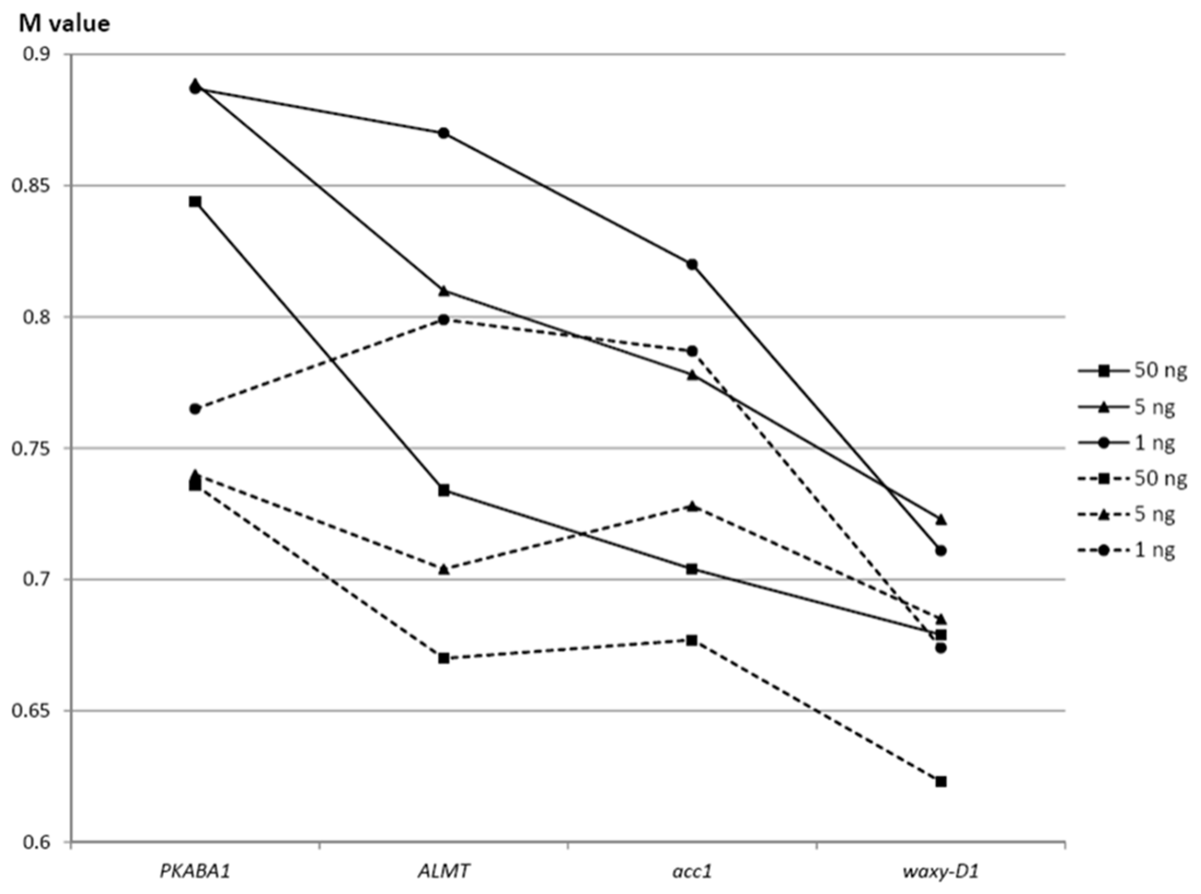
GeNorm analysis of four wheat endogenous reference genes assays among common wheat cultivars. Solid lines were results of analysis using all 39 cultivars, and dashed lines were results of analysis excluding the four cultivars with SNPs (Zhongguochun, Zhengmai 9023, Zhoumai 16, and Zimai 12).

To further evaluate the consistency of each real-time PCR assay, GeNorm analysis was performed using GeNorm software (version 3.5). M values of each reference gene in all lines were calculated. The highest M values were observed for *PKABA1* gene (0.884, 0.890, and 0.887 for 50 ng, 5 ng, and 1 ng genomic DNA, respectively), while *Waxy-D1* gene had the lowest M values (0.629, 0.723, and 0.711 for 50 ng, 5 ng, and 1 ng genomic DNA, respectively) (Figure **3**). In further analyses excluding the four lines with SNPs (Zhongguochun, Zhengmai 9023, Zhoumai 16, and Zimai 12), *Waxy-D1* assay still had the lowest M values (0.623, 0.685, and 0.674 for 50 ng, 5 ng, and 1 ng genomic DNA, respectively) (Figure **3**). Overall evaluation using calculated M values in GeNorm analysis suggested that the *Waxy-D1* real-time PCR assay was more consistent than the other assays and had the best PCR performance among all four assays. 

## Discussion

In order to develop GM wheat detection methods, including selection of proper wheat endogenous reference genes and their assays, researchers have reported several wheat endogenous reference genes and assays for GM wheat analysis in the past few years. However, there are still difficulties in selecting suitable endogenous reference genes for GM wheat analysis because of the complicated genotypes and karyotypes among wheat species ( *Triticum* L genus). Based on qualitative PCR amplification results, amplicons of *Acc1* gene and *PKABA1* gene were not only observed in all hexaploid common wheat lines, but also in *Aegilops* *speltoides* Tausch (BB), *Triticum* *urartu* L (AA), and durum wheat (BBAA), indicating that *Acc1* and *PKABA1* assays had low specificity for common wheat. *ALMT1* and *Waxy-D1* gene amplicons could not be amplified from *Aegilops* *speltoides* Tausch (BB), *Triticum* *urartu* L (AA), and durum wheat (BBAA), showing that these two genes were more suitable for identifying common wheat species. The results also showed the *ACC1* gene came from B genome, *PKABA1* from A genome, and the *Waxy-D1* and *ALMT1* genes from the D genome of wheat. Therefore, it was impossible to specifically identify the Chinese octoploid triticale from common wheat lines employing any gene from haploid A, B and D genome. These results demonstrated that the genotype and karyotype situations should be considered in selecting new endogenous reference genes for any given species.

The gene allele stability among different lines is another important parameter in validating and harmonizing the endogenous reference gene assays for GMO detection. It can be evaluated by target DNA sequencing/alignment and PCR performance analysis. Sequence alignment of target DNA from different wheat lines in this study revealed SNPs in several lines, such as one SNP of C to T in *Acc1* gene, two SNPs of A to C and G to A in *ALMT1* gene, and one SNP of G to A in *PKABA1* gene. It has been reported previously that SNPs in real-time PCR primer/probe annealing regions could affect PCR efficiency in quantification of GM contents, virus, and microorganisms [23–27]. In our study, increased *Ct* values (0.77<*ΔCt*<3.41) and decreased PCR efficiencies (0.12<*Δ*E<0.24) were observed in *Acc1*, *ALMT1*, and *PKABA1* assays when lines with SNPs in primer or probe region were tested. The SNPs effects on PCR efficiency were also confirmed by new real-time PCR assays employing re-designed homogenous primers or probes eliminating SNPs. These results demonstrated the SNPs could decrease the real-time PCR efficiency, which would result in an over-estimation of GM content in a GMO assay. Although previous reports have suggested that *Ct* variation for an endogenous reference gene assay among different lines might come from different qualities of extracted DNA, errors associated with DNA dilution, inaccurate measurement of DNA concentrations, or the calculation error between the copy number and DNA quantity because of the complex haploid genome size [19,20], our study indicated that the *Ct* differences of the four wheat endogenous reference gene assays among different lines could originate from different PCR performances. A collaborative ring trial validation of the selected real-time PCR assays might further improve the reliability and applicability of our results by considering the robustness of the assays in different laboratories, equipment, and brands of mastermix.

Based on the combined results of high species specificity, no SNPs in target sequence, and high PCR performance among different wheat lines, we concluded that *Waxy-D1* gene was the most suitable candidate among the four reported wheat endogenous reference genes, and its real-time PCR assay would be very useful in establishing accurate and creditable quantitative PCR assay for GM wheat.

## Supporting Information

File S1 **Supporting tables**.
Table S1, List of the 43 seed samples of *Triticum* genus and the specificity test results of the four endogenous reference genes. Table S2, Ct values of the 39 common wheat cultivars and 1 durum wheat cultivar from the four endogenous reference gene assays.
(DOCX)
