# Supplementary material for: Evaluation of Four Endogenous Reference Genes and Their Real-Time PCR Assays for Common Wheat Quantification in GMOs Detection
Source: PLoS One. 2013 Sep 30;8(9):e75850. doi: 10.1371/journal.pone.0075850 (PMC3786954; doi:10.1371/journal.pone.0075850)
Supplement: File S1 — Supporting tables. Table S1, List of the 43 seed samples of Triticum genus and the specificity test results of the four endogenous reference genes. Table S2, Ct values of the 39 common wheat cultivars and 1 durum wheat cultivar from the four endogenous reference gene assays. (DOCX) [file pone.0075850.s001.docx]

**File S1: Supporting tables**

**Table S1.** List of the 43 seed samples of *Triticum* genus and the specificity test results of the four endogenous reference genes.

| **Genotype** | **Chromosome number** | **Cultivar Name** | **Origin** | ***acc1*** | ***ALMT*** | ***waxy-D1*** | ***PKABA1*** |
| --- | --- | --- | --- | --- | --- | --- | --- |
| D | 2n=14 | *Aegilops tauschii* |  | NA | P | P | NA |
| B | 2n=14 | *Ae.speltoides Tausch* |  | P | NA | NA | NA |
| A | 2n=14 | *Triticum urartu Thum.ex Gandil* |  | NA | NA | NA | P |
| BA | 2n=28 | Cannizzo (*Triticum turgidum*) | Italy | P | NA | NA | P |
| BAD | 2n=42 | Wanmai 52 | Anhui | P | P | P | P |
|  |  | Jindong 8 | Beijing | P | P | P | P |
|  |  | Jing 411 | Beijing | P | P | P | P |
|  |  | Lunxuan 987 | Beijing | P | P | P | P |
|  |  | Zhongmai 175 | Beijing | P | P | P | P |
|  |  | Han 6172 | Hebei | P | P | P | P |
|  |  | Hengguan 35 | Hebei | P | P | P | P |
|  |  | Kelong 199 | Hebei | P | P | P | P |
|  |  | Shi 4185 | Hebei | P | P | P | P |
|  |  | shijiazhuang 8 | Hebei | P | P | P | P |
|  |  | Shimai 15 | Hebei | P | P | P | P |
|  |  | Shixin 733 | Hebei | P | P | P | P |
|  |  | Shiyou 20 | Hebei | P | P | P | P |
|  |  | Yishi 2-1 | Hebei | P | P | P | P |
|  |  | Kefeng 12 | Heilongjiang | P | P | P | P |
|  |  | Kenjiu 10 | Heilongjiang | P | P | P | P |
|  |  | Longfumai 18 | Heilongjiang | P | P | P | P |
|  |  | Longmai 30 | Heilongjiang | P | P | P | P |
|  |  | Aikang 58 | Henan | P | P | P | P |
|  |  | Luohan 2 | Henan | P | P | P | P |
|  |  | Xinmai 18 | Henan | P | P | P | P |
|  |  | Yanzhan 4110 | Henan | P | P | P | P |
|  |  | Yumai 49 | Henan | P | P | P | P |
|  |  | Zhengmai 9023 | Henan | P | P | P | P |
|  |  | Zhoumai 16 | Henan | P | P | P | P |
|  |  | Ningchun 50 | Ningxia | P | P | P | P |
|  |  | Jimai 19 | Shandong | P | P | P | P |
|  |  | Lumai 14 | Shandong | P | P | P | P |
|  |  | Weimai 8 | Shandong | P | P | P | P |
|  |  | Yannong 19 | Shandong | P | P | P | P |
|  |  | Zimai 12 | Shandong | P | P | P | P |
|  |  | Chang 6878 | Shanxi | P | P | P | P |
|  |  | Jinmai 47 | Shanxi | P | P | P | P |
|  |  | Linfeng 3 | Shanxi | P | P | P | P |
|  |  | Xinong 987 | Shanxi | P | P | P | P |
|  |  | Yuanfeng 175 | Shanxi | P | P | P | P |
|  |  | Xindong 28 | Xinjiang | P | P | P | P |
| BADR | 2n=56 | Xiaoyan 22 | Shanxi | P | P | P | P |
|  |  | Zhongguochun | Beijing | P | P | P | P |

“P” means positive result, and “NA” means negative result.

**Table S2. Ct values of the 39 common wheat cultivars and 1 durum wheat cultivar from the four endogenous reference gene assays.**

| **Cultivar Name** | **50ng** | | | |  | **5ng** | | | |  | **1ng** | | | |
| --- | --- | --- | --- | --- | --- | --- | --- | --- | --- | --- | --- | --- | --- | --- |
|  | **acc1** | **ALMT** | **waxy-D1** | ***PKABA1*** |  | **acc1** | **ALMT** | **waxy-D1** | ***PKABA1*** |  | **acc1** | **ALMT** | **waxy-D1** | ***PKABA1*** |
| Wanmai 52 | 23.65 | 28.9 | 29.68 | 28.44 |  | 26.76 | 31.24 | 33.06 | 31.57 |  | 27.83 | 34.6 | 35.12 | 34.06 |
| Jindong 8 | 23.78 | 28.7 | 29.65 | 27.66 |  | 28.49 | 32.07 | 33.07 | 31.05 |  | 29.58 | 34.73 | 35.52 | 33.68 |
| Jing 411 | 23.72 | 28.69 | 29.45 | 29.38 |  | 27.36 | 31.99 | 32.56 | 32.55 |  | 29.88 | 34.45 | 35.03 | 34.86 |
| Lunxuan 987 | 24.67 | 28.58 | 29.77 | 27.94 |  | 27.83 | 32.06 | 33.11 | 31.07 |  | 30.37 | 34.86 | 35.32 | 33.48 |
| Zhongmai 175 | 23.26 | 28.89 | 29.82 | 29.64 |  | 28.2 | 32.33 | 32.84 | 32.33 |  | 30.05 | 34.62 | 35.19 | 34.79 |
| Han 6172 | 23.54 | 29.14 | 29.95 | 27.87 |  | 26.92 | 32.57 | 33.09 | 30.73 |  | 29.14 | 34.88 | 35.16 | 33.08 |
| Hengguan 35 | 24.4 | 27.36 | 29.83 | 29.19 |  | 27.29 | 30.77 | 33.63 | 31.9 |  | 30.5 | 33.19 | 36.07 | 34.03 |
| Kelong 199 | 23.77 | 28.18 | 29.56 | 29.38 |  | 26.87 | 31.56 | 33.08 | 32.45 |  | 29.03 | 33.84 | 35.6 | 34.72 |
| Shi 4185 | 23.83 | 28 | 29.73 | 28.31 |  | 27.67 | 31.11 | 32.95 | 31.47 |  | 29.29 | 33.95 | 35.25 | 33.99 |
| shijiazhuang 8 | 24.31 | 28.79 | 29.6 | 28.85 |  | 27.2 | 32.24 | 33.17 | 31.27 |  | 29.61 | 34.59 | 35.35 | 33.39 |
| Shimai 15 | 24.04 | 27.73 | 30 | 28 |  | 27.67 | 31.15 | 33.36 | 31.42 |  | 29.82 | 33.82 | 35.26 | 34.46 |
| Shixin 733 | 24.1 | 28.53 | 30.51 | 27.84 |  | 27.51 | 31.75 | 33.58 | 31.25 |  | 29.74 | 34.15 | 35.81 | 34.01 |
| Shiyou 20 | 23.09 | 28.97 | 29.66 | 29.23 |  | 26.56 | 32.45 | 33.31 | 32.43 |  | 28.41 | 34.89 | 35.54 | 34.69 |
| Yishi 2-1 | 23.71 | 28.49 | 30.69 | 28.21 |  | 26.96 | 32.02 | 34.13 | 31.53 |  | 29.16 | 35 | 36.27 | 34.06 |
| Kefeng 12 | 23.63 | 28.73 | 29.54 | 28.84 |  | 26.82 | 32.05 | 32.84 | 32.2 |  | 29.26 | 34.48 | 34.91 | 34.82 |
| Kenjiu 10 | 24.65 | 28.92 | 29.22 | 28.63 |  | 27.23 | 32.32 | 32.94 | 31.51 |  | 29.42 | 34.71 | 34.89 | 33.84 |
| Longfumai 18 | 23.61 | 28.52 | 29.21 | 27.7 |  | 27.04 | 31.9 | 32.67 | 30.98 |  | 29.19 | 34.48 | 35.21 | 33.55 |
| Longmai 30 | 23.93 | 28.28 | 29.03 | 28.01 |  | 25.97 | 31.62 | 32.34 | 31.22 |  | 28.37 | 34.19 | 35.11 | 33.52 |
| Aikang 58 | 23.41 | 28.34 | 29.82 | 29.05 |  | 26.63 | 31.75 | 33.22 | 32.2 |  | 29.08 | 33.95 | 35.43 | 34.81 |
| Luohan 2 | 24.23 | 28.42 | 30.06 | 28.63 |  | 27.29 | 31.35 | 33.32 | 31.58 |  | 29.34 | 35.74 | 36.46 | 34.12 |
| Xinmai 18 | 23.83 | 28.12 | 29.17 | 27.97 |  | 27.31 | 31.79 | 32.6 | 31.26 |  | 29.11 | 34.87 | 34.78 | 33.76 |
| Yanzhan 4110 | 24.28 | 29.2 | 29.98 | 29.63 |  | 27.58 | 32.65 | 33.29 | 32.34 |  | 29.99 | 35.05 | 35.66 | 34.64 |
| Yumai 49 | 24.59 | 28.56 | 29.01 | 28.83 |  | 27.47 | 32.13 | 32.45 | 31.83 |  | 29.71 | 34.4 | 34.78 | 33.77 |
| Zhengmai 9023 | 23.69 | 29.34 | 29.47 | 28.87 |  | 27.61 | 32.89 | 32.84 | 31.14 |  | 29.63 | 35.93 | 35.02 | 33.21 |
| Zhoumai 16 | 23.34 | 27.38 | 29.5 | 30.29 |  | 26.54 | 30.95 | 33.34 | 33.39 |  | 29.27 | 33.36 | 36.07 | 35.4 |
| Ningchun 50 | 23.08 | 28.54 | 29.04 | 27.79 |  | 26.27 | 32 | 32.41 | 31.16 |  | 28.47 | 34.48 | 34.69 | 33.64 |
| Jimai 19 | 24.42 | 28.45 | 29.85 | 29.93 |  | 28.1 | 31.81 | 32.27 | 32.66 |  | 30.24 | 34.21 | 34.64 | 34.78 |
| Lumai 14 | 23.39 | 29.21 | 29.75 | 28.71 |  | 26.42 | 32.64 | 33.32 | 31.83 |  | 28.64 | 34.94 | 35.46 | 34.48 |
| Weimai 8 | 24.27 | 28.64 | 29.8 | 28.66 |  | 27.13 | 31.93 | 32.96 | 30.69 |  | 29.28 | 34.31 | 35.16 | 33.11 |
| Yannong 19 | 23.38 | 28.22 | 29.75 | 28.12 |  | 26.65 | 31.88 | 33.05 | 30.66 |  | 28.83 | 34.22 | 35.38 | 33.8 |
| Zimai 12 | 24.42 | 29.05 | 29.14 | 30.27 |  | 27.16 | 31.58 | 33.57 | 33.54 |  | 28.92 | 34.88 | 35.81 | 35.41 |
| Chang 6878 | 23.96 | 27.9 | 29.29 | 27.87 |  | 27.26 | 31.21 | 32.74 | 30.95 |  | 29.22 | 33.65 | 35.07 | 33.27 |
| Jinmai 47 | 22.77 | 28.64 | 29.45 | 28.75 |  | 26.35 | 32.02 | 32.83 | 31.69 |  | 28.46 | 34.45 | 35.18 | 34 |
| Linfeng 3 | 23.47 | 27.7 | 30.35 | 28.69 |  | 26.8 | 31.09 | 33.84 | 31.79 |  | 28.71 | 33.52 | 35.74 | 34.04 |
| Xinong 987 | 24.04 | 28.12 | 29.34 | 28.25 |  | 27.6 | 31.66 | 33 | 31.39 |  | 29.64 | 34.07 | 35.09 | 33.07 |
| Yuanfeng 175 | 23.46 | 27.7 | 29.37 | 28.5 |  | 26.99 | 31.09 | 32.98 | 31 |  | 29.23 | 33.52 | 35.25 | 33.26 |
| Xindong 28 | 24.49 | 27.9 | 30 | 29.47 |  | 27.42 | 31.32 | 33.25 | 32.27 |  | 29.24 | 32.72 | 35.35 | 34.29 |
| Xiaoyan 22 | 23.65 | 28.66 | 30.24 | 28.45 |  | 26.79 | 32.04 | 33.83 | 31.06 |  | 28.79 | 35.43 | 36.02 | 33.54 |
| Zhongguochun | 23.77 | 29.49 | 30.09 | 28.52 |  | 26.57 | 32.95 | 33.05 | 30.86 |  | 28.6 | 35.25 | 35.28 | 32.49 |
| Cannizzo (durum wheat) | 26.29 | NA* | NA* | 27.24 |  | 28.68 | NA* | NA* | 30.75 |  | 30.78 | NA* | NA* | 32.88 |

“NA” means no data was obtained.
